# Essential pre-pregnancy and pregnancy interventions for improved maternal, newborn and child health

**DOI:** 10.1186/1742-4755-11-S1-S2

**Published:** 2014-08-21

**Authors:** Zohra S Lassi, Tarab Mansoor, Rehana A Salam, Jai K Das, Zulfiqar A Bhutta

**Affiliations:** 1 Division of Women and Child Health, Aga Khan University, Karachi, Pakistan

**Keywords:** preconception, prepregnancy, pregnancy, essential interventions

## Abstract

The statistics related to pregnancy and its outcomes are staggering: annually, an estimated 250000-280000 women die during childbirth. Unfortunately, a large number of women receive little or no care during or before pregnancy. At a period of critical vulnerability, interventions can be effectively delivered to improve the health of women and their newborns and also to make their pregnancy safe. This paper reviews the interventions that are most effective during preconception and pregnancy period and synergistically improve maternal and neonatal outcomes. Among pre-pregnancy interventions, family planning and advocating pregnancies at appropriate intervals; prevention and management of sexually transmitted infections including HIV; and peri-conceptual folic-acid supplementation have shown significant impact on reducing maternal and neonatal morbidity and mortality. During pregnancy, interventions including antenatal care visit model; iron and folic acid supplementation; tetanus Immunisation; prevention and management of malaria; prevention and management of HIV and PMTCT; calcium for hypertension; anti-Platelet agents (low dose aspirin) for prevention of Pre-eclampsia; anti-hypertensives for treating severe hypertension; management of pregnancy-induced hypertension/eclampsia; external cephalic version for breech presentation at term (>36 weeks); management of preterm, premature rupture of membranes; management of unintended pregnancy; and home visits for women and children across the continuum of care have shown maximum impact on reducing the burden of maternal and newborn morbidity and mortality. All of the interventions summarized in this paper have the potential to improve maternal mortality rates and also contribute to better health care practices during preconception and periconception period.

## Introduction

Although maternal mortality has decreased during the past decade, the proportion of births attended by a skilled health personnel still remains 68% in developing regions, often leading to tragic consequences [1]. Annually, an estimated 250000-280000 women die during childbirth [2] while over 15 million are chronically afflicted with complications which occur during pregnancy [3]. Adressing these issues in developing regions are critical forimproving maternal and neonatal health status and decreasing the associated morbidity and mortality.

In order to attain optimum level of health during pregnancy and the best possible maternal and neonatal health outcomes, effective interventions need to be delivered during preconception period and throughout pregnancy. In recent years, there has been increaased awareness regarding the importance of preconception period and efforts have been made to increase awareness and promote health right from childhood and adolescence onwards [4-12]. Research has also established linkages of preconception interventions with improved maternal, perinatal and neonatal health outcomes and it has been suggested that several proven interventions recommended during pregnancy may be even more effective if implemented before conception. On the other hand, interventions delivered during pregnancy also play a definitive beneficial role for improved maternal, fetal and neonatal health and survival. Maternal undernutrition, infections and chronic diseases carry a high risk for poor maternal and neonatal health and therefore, regular anatenatal checkups with improved screening can save many maternal and neonatal lives.

The motive of this review is to highlight the effectiveness of essential pre-pregnancy and pregnancy interventions that have an alleged impact on maternal, fetal and newborn outcomes and can be suitably delivered to low and middle income countries (LMIC) where minimal essential care is generally available.

## Methodology

The methodology has been described in detail elsewhere [13]. In short, the review included all pre-pregnancy and pregnancy interventions based on current World Health Organization (WHO) guidelines and recent Lancet series which have an alleged impact on reducing maternal, neonatal and child mortality; suitable for delivery in LMICs, and those that can be delivered through the health sector (community level up to the referral level of health care) (Table 1). All relevant pre-pregnancy and pregnancy intervention reviews were identified from the electronic databases and were classified according to evidence and delivery strategies. Search was conducted in the following electronic databases: the Cochrane database of systematic reviews, the Cochrane database of abstract reviews of effectiveness (DARE), theCochrane database of systematic reviews of randomized control trials (RCT’s), and PubMed. The reference lists of the reviews and recommendations from experts in the field were also used as sources to obtain additional publications. The principal focus was on the existing systematic reviews and meta-analysis. Based on the efficiency of the interventions, these were then classisifed in categories from A to E (where A signified strongly beneficial effect while E indicated harmful effect) on different levels of health sector (community/outreach/referral).

**Table 1:** Characteristics of the included reviews on pre-pregnancy and pregnancy interventions

Reviews	Objective	Type of Studies included (number)	Cochrane/non-Cochrane	Pooled Data (Y/N)	Outcomes reported
**Conde-Agudelo 2007 **[20]	To explore the association between birth spacing and risk of adverse maternal outcomes	Observational studies = 22	Non-Cochrane	Yes	Pre-eclampsia, maternal outcomes
**Conde-Agudelo 2007 **[19]	To examine the association between birth spacing and relative risk of adverse perinatal outcomes.	Observational studies = 67	Non-Cochrane	Yes	preterm birth, low birth weight, and small for gestational age
**Kozuki 2013 **[21]	To examine the association between short/long birth intervals and adverse neonatal outcomes by calculating and meta-analyzing associations using original data from cohort studies conducted in low-and middle-income countries.	Cohort =5	Non-cohrane	Yes	Small for gestational age, Infant mortality, Preterm births
**Anglemyer 2013 **[23]	To determine if ART use in an HIV-infected member of an HIV-discordant couple is associated with lower risk of HIV transmission to the uninfected partner compared to untreated discordant couples.	Observational studies = 7	Cochrane	Yes	Episodes of HIV transmisison, index partner's CD4 cell count.
**Ng 2011 **[24]	To determine the impact of population-based biomedical STI interventions on the incidence of HIV infection.	RCT: 4	Cochrane	Yes	incident HIV infection, prevalence of syphilis
**Blencowe 2010 **[29]	To review the evidence for, and estimate the effect of, folic acid fortification/supplementation on neonatal mortality due to NTDs, especially in low-income countries.	RCT: 3 Observational studies: 8	Non-Cochrane	Yes	NTD recurrence, NTD incidence, congenital abnormalities, neonatal deaths
**De-Regil 2010 **[30]	This review examined whether folate supplementation before and during early pregnancy can reduce neural tube and other birth defects (including cleft palate) without causing adverse outcomes for mothers or babies.	RCTs: 5	Cochrane	Yes	Prevention of NTDs, incidence of NTDs, reoccurrence of NTDs, cleft palate, cleft lip, congenital cardiovascular defects, miscarriages or any other birth defects.
**Imdad 2011 **[31]	To evaluate the effectiveness of peri-conceptional folic acid supplementation in reducing neural tube defects (NTD), related stillbirths and balanced protein energy and multiple micronutrients supplementation during pregnancy in reducing all-cause stillbirths.	RCTs: 18	Non-Cochrane	Yes	NTDs, stillbirths
**Carrolli 2001 **[33]	a systematic review of randomised trials assessing the effectiveness of different models of antenatal care.	RCTs: 7	Non-Cochrane	Yes	pre-eclampsia, urinary-tract infection, postpartum anaemia, maternal mortality, low birth weight
**Dowswell 2010 **[34]	To compare the effects of antenatal care programmes with reduced visits for low-risk women with standard care.	RCTs: 7	Cochrane	Yes	Perinatal mortality, admission to neonatal intensive care
**Homer 2012 **[35]	The first objective was to compare the effects of group antenatal care versus one-to-one care on outcomes for women and their babies.	RCTs and q-RCTs : 2	Cochrane	Yes	Preterm birth, low birth weight, small-for-gestational age and perinatal mortality.
**Pena-Rosas 2012 **[41]	To assess the effects of daily oral iron supplements for pregnant women, either alone or in conjunction with folic acid, or with other vitamins and minerals as a public health intervention.	RCTs and q-RCTs : 43	Cochrane	Yes	low birthweight, mean birth weight, maternal anaemia, iron deficiency at term, side effects, haemoglobin (Hb) concentrations
**Yakoob 2011 **[42]	To address the impact of iron with and without folate supplementation on maternal anemia and provides outcome specific quality according to the Child Health Epidemiology Reference Group (CHERG) guidelines.	RCTs and q-RCTs : 31	Non-Cochrane	Yes	incidence of anemia at term, iron deficiency anemia at term
**Lassi 2013 **[43]	To assess the effectiveness of oral folic acid supplementation alone or with other micronutrients versus no folic acid (placebo or same micronutrients but no folic acid) during pregnancy on haematological and biochemical parameters during pregnancy and on pregnancy outcomes.	RCTs and q-RCTs : 31	Cochrane	Yes	Preterm birth, stillbirths/neonatal deaths, mean birthweight, anaemia, mean pre-delivery haemoglobin level, mean pre-delivery serum folate levels, mean pre-delivery red cell folate levels, incidence of megaloblasticanaemia
**Demicheli 2013 **[47]	To assess the effectiveness of tetanus toxoid, administered to women of childbearing age or pregnant women, to prevent cases of, and deaths from, neonatal tetanus	RCTs: 2	Non-Cochrane	No	vaccine effectiveness was 43%
**Blencowe 2010 **[48]	To review the evidence for and estimate the effect on neonatal tetanus mortality of immunization with tetanus toxoid of pregnant women, or women of childbearing age.	RCT: 1 CT: 1	Non-Cochrane	Yes	mortality from neonatal tetanus
**Garner 2006 **[52]	To assess drugs given to prevent malaria infection and its consequences in pregnant women living in malarial areas. This includes prophylaxis and intermittent preventive treatment (IPT).	RCTs and q-RCTs : 16	Cochrane	Yes	antenatal parasitaemia, placental malaria, perinatal deaths
**TerKuile 2007 **[53]	To determine the effect of increasing resistance to sulfadoxine-pyrimethamine on the efficacy of IPT during pregnancy in Africa.	RCTs: 4	Non-Cochrane	Yes	placental malaria, low birth weight, anemia
**Lengeler 2004 **[54]	To assess the impact of insecticide-treated bed nets or curtains on mortality, malarial illness (life-threatening and mild), malaria parasitaemia, anaemia, and spleen rates.	RCTs: 22	Cochrane	Yes	protective efficacy, severe malaria, parasite prevalence, high parasitaemia, splenomegaly (30% PE), haemoglobin
**Gamble 2007 **[55]	To compare the impact of ITNs with no nets or untreated nets on preventing malaria in pregnancy	RCTs: 5	Non-Cochrane	Yes	low birthweight, stillbirths/abortions in the first to fourth pregnancy
**Gamble 2006 **[56]	To compare the impact of ITNs with no nets or untreated nets on preventing malaria in pregnancy.	RCTS: 6	Cochrane	Yes	low birthweight, stillbirths/abortions in the first to fourth pregnancy
**Eisele 2010 **[57]	To estimate the effect of ITNs and IRS on preventing malaria-attributable mortality in children 1–59 months, and to estimate the effect of ITNs and IPTp on preventing neonatal and child mortality through improvements in birth outcomes.	RCTs: 14	Non-Cochrane	Yes	rotective efficacy, malaria-attributable mortality 1–59 months, prevention interventions in pregnancy
**Lumley 2009 **[58]	To assess the effects of smoking cessation interventions during pregnancy on smoking behaviour and perinatal health outcomes.	RCTs: 72	Cochrane	Yes	reduction in smoking in late pregnancy, relapse
**Coleman 2012 **[59]	To determine the efficacy and safety of smoking cessation pharmacotherapies, including NRT, varenicline and bupropion (or any other medications) when used to support smoking cessation in pregnancy.	RCTs: 6	Cochrane	Yes	smoking cessation in later pregnancy
**Blencowe 2011 **[61]	This review sought to estimate the effect of detection and treatment of active syphilis in pregnancy with at least 2.4MU benzathine penicillin (or equivalent) on syphilis-related stillbirths and neonatal mortality.	Observational studies: 25	Non-Cochrane	Yes	Stillbirth, preterm delivery, neonatal deaths
**Walker 2001 **[62]	To identify the most effective antibiotic treatment regimen (in terms of dose, length of course and mode of administration) of syphilis with and without concomitant infection with HIV for pregnant women infected with syphilis.	RCTs and q-RCTs : 26	Cochrane	No	None matched predetermined criteria for comparison
**Wiysonge 2011 **[65]	To assess the effects of antenatal and intrapartum vitamin A supplementation on the risk of MTCT of HIV infection and infant and maternal mortality and morbidity, and the tolerability of vitamin A supplementation.	RCTs: 4	Cochrane	Yes	MTCT of HIV infection, birth weight, stillbirths, preterm births, death by 24 months among live births
**Shey 2002 **[66]	To estimate the effect of vaginal lavage on the risk of MTCT of HIV and infant and maternal mortality and morbidity, as well as tolerability of vaginal lavage in HIV infected women.	RCT: 1	Cochrane	No	vaginal disinfection on MTCT of HIV
**Kesho Bora 2009 **[64]	Triple-antiretroviral (ARV) prophylaxis during pregnancy and breastfeeding compared to short-ARV prophylaxis to prevent mother-to-child transmission of HIV-1 (PMTCT): the Kesho Bora randomized controlled clinical trial in five sites in Burkina Faso, Kenya	1 study in five different location	Non Cochrane	No	Extended triple ARV regimen consisting of the anti-HIV drugs zidovudine, lamivudine andlopinavir/ritonavir, from the last trimester of pregnancy and continued during breastfeeding up to the age of six months.
**Imdad 2011 **[78]	To evaluate preventive effect of calcium supplementation during pregnancy on gestational hypertensive disorders and related maternal and neonatal mortality in developing countries.	RCTs: 10	Non-Cochrane	Yes	gestational hypertension, pre-eclampsia, neonatal mortality
**Hofmeyr 2010 **[79]	To assess the effects of calcium supplementation during pregnancy on hypertensive disorders of pregnancy and related maternal and child outcomes.	RCTs: 13	Cochrane	Yes	high blood pressure, pre-eclampsia, preterm birth, stillbirth or death before discharge from hospital, maternal death or serious morbidity
**Jabeen 2011 **[81]	To review the effect of aspirin, calcium supplementation, antihypertensive agents and magnesium sulphate on risk stillbirths.	RCTs: 82	Non-Cochrane	Yes	stillbirths
**Duley 2013 **[83]	To assess the effectiveness and safety of antiplatelet agents for women at risk of developing pre-eclampsia.	RCTs: 59	Cochrane yes	Yes	pre-eclampsia, maternal risk, preterm birth, fetal or neonatal deaths, small-for-gestational age babies
**Askie 2007 **[84]	to assess the use of antiplatelet agents for the primary prevention of pre-eclampsia, and to explore which women are likely to benefit most.	RCTs: 31	Non-Cochrane	Yes	pre-eclampsia, of delivering before 34 weeks,serious adverse outcome
**Duley 2013 **[88]	To compare different antihypertensive drugs for very high blood pressure during pregnancy.	RCTs: 24	Cochrane	Yes	persistent high blood, risk of HELLP, risk of hypotension, eclampsia, respiratory difficulties, but fewer side-effects, less postpartum haemorrhage
**Magee 2003 **[89]	to assess whether oral beta-blockers are overall better than placebo, or no beta-blocker, for women with mild-moderate hypertension during pregnancy, and to assess whether oral beta-blockers have any advantages over other antihypertensive agents for women with mild-moderate hypertension during pregnancy.	RCTs; 27	Cochrane	Yes	Both maternal outcomes (e.g., the incidence of severe hypertension) and perinatal outcomes
**Duley 2010 **[90]	The objective of this review was to assess the effects of magnesium sulphate compared with diazepam when used for the care of women with eclampsia. Magnesium sulphate is compared with phenytoin and with lytic cocktail in other Cochrane reviews.	RCTs: 7	Cochrane	Yes	Recurrence of seizures, maternal morbidity, perinatal mortality, neonatal mortality, Apgar score
**Duley 2010 **[91]	The objective of this review was to assess the effects of magnesium sulphate compared with phenytoin when used for the care of women with eclampsia.	RCTs: 7	Cochrane	Yes	Recurrence of seizures, maternal morbidity, perinatal mortality, neonatal mortality, Apgar score
**Duley 2010 **[92]	To assess the effects of magnesium sulphate, and other anticonvulsants, for prevention of eclampsia.	RCTs: 15	Cochrane	Yes	Eclampsia, maternal death, serious maternal morbidity, placental abruption, caesarean section, stillbirths
**Duley 2010 **[93]	To assess the effects of magnesium sulphate compared with lytic cocktail (usually chlorpromazine, promethazine and pethidine) when used for the care of women with eclampsia	RCTs: 3	Cochrane	Yes	maternal deaths, seizures, respiratory depression , coma, pneumonia
**Cluver 2012 **[95]	To assess interventions such as tocolysis, fetal acoustic stimulation, regional analgesia, transabdominalamnioinfusion or systemic opioids on ECV for a breech baby at term.	RCTs and qRCTs: 25	Cochrane	Yes	cephalic presentations in labour, caesarean sections
**Hutton 2006 **[96]	To assess the effectiveness of a policy of beginning ECV before term (before 37 weeks' gestation) for breech presentation on fetal presentation at birth, method of delivery, and the rate of preterm birth, perinatal morbidity, stillbirth or neonatal mortality.	RCTs: 3	Cochrane	No	non-cephalic presentation at birth
**Hofmeyr 2012 **[97]	The objective of this review was to assess the effects of postural management of breech presentation on measures of pregnancyoutcome.We evaluated procedures in which the motherrests with herpelvis elevated. These include the knee-chestposition, and a supin e position with the pelvis elevated with a wedge-shaped cushion	RCTs: 6	Cochrane	Yes	non-cephalic births, Cesarean section and Apgar scores below 7 at one minute, r
**Hofmeyr 2012 **[98]	The objective of this review was to assess the effects of ECV at or near term on measures of pregnancy outcome. Methods of facilitatingECV, and ECV before term are reviewed separately	RCTs: 7	Cochrane	Yes	non-cephalic presentation at birth, Cesarean section
**Hofmeyr 2003 **[99]	To assess the effects of planned caesarean section for singleton breech presentation at term on measures of pregnancy outcome.	RCTs: 3	Cochrane	Yes	Caesarean delivery, perinatal or neonatal death or serious neonatal morbidity, urinary incontinence, abdominal pain , perineal pain
**Coyle 2012 **[100]	To examine the effectiveness and safety of moxibustion on changing the presentation of an unborn baby in the breech position, the need for external cephalic version (ECV), mode of birth, and perinatal morbidity and mortality for breech presentation.	RCTs: 3	Cochrane	Yes	need for ECV, use of oxytocin before or during
**Buchanan 2010 **[102]	To assess the effect of planned early birth compared with expectant management for pregnancies complicated with PPROM prior to 37 weeks' gestation.	RCTs: 7	Cochrane	Yes	neonatal sepsis, respiratory distress, incidence of caesarean section
**Kenyon 2010 **[103]	To evaluate the immediate and long-term effects of administering antibiotics to women with pROM before 37 weeks, on maternal infectious morbidity, fetal and neonatal morbidity and mortality, and longer term childhood development.	RCTs: 19	Cochrane	Yes	Chorioamnionitis, neonatal morbidity, neonatal infection, use of surfactant
**Cousens 2010 **[104]	To review the evidence for and estimate the effect on neonatal mortality due to pre-term birth complications or infection, of administration of antibiotics to women with pPROM, in low and middle-income countries.	RCTs: 18	Non-Cochrane	Yes	respiratory distress syndrome , early onset postnatal infection, neonatal mortality
**Roberts 2006 **[105]	To assess the effects on fetal and neonatal morbidity and mortality, on maternal mortality and morbidity, and on the child in later life of administering corticosteroids to the mother before anticipated preterm birth.	RCTs: 22	Cochrane	Yes	chorioamnionitis or puerperal sepsis, neonatal death, RDS, cerebroventricularhaemorrhage, necrotisingenterocolitis
**Mwansa-Kambafwile 2010 **[108]	To review the evidence for and estimate the effect on cause-specific neonatal mortality of administration of antenatal steroids to women with anticipated preterm labour, with additional analysis for the effect in low- and middle-income countries.	Studies: 44 RCTs: 18	Non-Cochrane	Yes	neonatal mortality among preterm infant
**Brownfoot 2008 **[109]	To assess the effects of different corticosteroid regimens for women at risk of preterm birth.	RCTs: 10	Cochrane	Yes	Incidence of intraventricularhaemorrhage, respiratory distress syndrome, bronchopulmonary dysplasia, severe intraventricularhaemorrhage, periventricular leukomalacia, perinatal death, or mean birthweight.
**WHO 2003 **[113]	evidence profiles related to the prioritized questions were prepared, based upon recent systematic reviews, most of which are included in the Cochrane Database of Systematic Reviews	-	-	-	-
**Kidney 2009 **[116]	The objective was to provide a systematic review of the effectiveness of community-level interventions to reduce maternal mortality.	RCTs: 5 Cohort: 8	Non-Cochrane	Yes	Maternal mortality
**Lassi 2010 **[117]	To assess the effectiveness of community-based intervention packages in reducing maternal and neonatal morbidity and mortality; and improving neonatal outcomes.	RCTs and qRCTs: 18	Cochrane	Yes	Maternal mortality, neonatal mortality, perinatal morality, stillbirths, newborn care practices
**Gogia 2010 **[118]	To determine whether home visits for neonatal care by community health workers can reduce infant and neonatal deaths and stillbirths in resource-limited settings.	RCTs: 5	Non-Cochrane	Yes	Neonatal death and stillbirth, and a significant improvement in antenatal and neonatal practice indicators (> 1 antenatal check-up, 2 doses of maternal tetanus toxoid, clean umbilical cord care, early breastfeeding and delayed bathing).
**Bhutta 2009 **[115]	examines the evidence for community and health systems approaches to improve uptake and quality of antenatal and intrapartum care,	RCTs: 9	Non-Cochrane	Yes	Stillbirths

## Results

### Pre-pregnancy interventions

#### Family planning

There is a large unmet need for family planning as at least 200 million women desire safe and effective family planning methods.An estimated 21.6million unsafe abortions took place worldwide in 2008, almost all in LMICs. Numbers of unsafe abortions have increased from 19.7 million in 2003 although the overall unsafe abortion rate remains unchanged at about 14 unsafe abortions per 1000 women aged 15–44 years [14]. Family planning prevents unwanted pregnancies and eliminates recourse to abortions. Both short and long inter-pregnancy intervals are associated with adverse perinatal outcomes [15-17] such as preterm birth, low birth weight (LBW), small for gestational age (SGA) and perinatal death. This link, however, may be confounded by different maternal characteristics and socio-economic status. To prevent these adverse events, birth spacing may be considered an intervention, especially in the developing world. Family planning is to plan when to have children and the use of birth control to delay pregnancy, including other techniques to implement such plans. Family planning services are defined as educational, comprehensive medical or social activities which enable individuals, including minors, to determine freely the number and spacing of their children and to select the means by which this may be achieved.

Short inter-pregnancy interval (IPIs) (less than 6 months) is associated with higher risk of preterm birth (odds ratio (OR) 1.40; 95% confidence interval (CI): 1.24, 1.58), LBW (OR 1.61; 95% CI: 1.39, 1.86), fetal death (OR 1.54, 95% CI: 1.06, 1.96) and SGA (OR 1.26; 95% CI: 1.18, 1.33) compared to IPI of 18 to 23 months. The risk is also significantly increased for these three adverse outcomes if the infant is conceived 60 months or more after a birth (preterm birth: OR 1.20; 95% CI: 1.17, 1.24; LBW: OR 1.43; 95% CI: 1.27, 1.62; fetal death OR 1.21, 95% CI: 1.07, 1.31 and SGA: OR 1.29; 95% CI: 1.20, 1.39) [18-20]. A recently published review with modified interval window reported the similar findings [21]. Birth interval of shorter than 18 months was associated with increased odds of SGA (OR 1.51, 95% CI: 1.31, 1.75), preterm (OR: 1.58, 95% CI: 1.19, 2.10) and infant mortality (OR 1.83, 95% CI: 1.19, 2.81) as compared to IPI of 36 to <60 months. On the other hand, birth interval over 60 months had increased risk of SGA (OR: 1.22, 95% CI: 1.07, 1.39) and term-SGA (OR: 1.14, 95% CI: 1.03, 1.27) when compared with IPI of 36 to <60 months. The efficacy and effectiveness for different methods of contraception were also compared. The efficacy of the withdrawal method was found to be maximum (4%) and the least one was for the implant (0.1%), while the effectiveness was 21.6% for periodic abstinence and 15.1% for withdrawal [22]. These findings suggest that short IPIs (less than 6 months) are associated with a significantly higher risk of preterm birth, LBW, fetal death and SGA infants compared to IPIs of 18 to 23 months. A recent review also reported higher risk of SGA, preterm births and infant mortality with IPIs of less than 18 months compared to 36 to <60 months. However, the risks were also higher if the infant is conceived 60 months or more after a birth. It is therefore advisable to plan pregnancies at appropriate intervals.

#### Prevention and management of sexually transmitted infections (STIs) including HIV

HIV was first declared as a pandemic in the early 1980s and the virus has become the leading cause of mortality across the globe, and the number of people it has infected and killed worldwide is astonishing. HIV is mainly spread in the population through sexual intercourse, needle sharing, blood transfusion or vertical transmission, which is the primary way by which children are infected. We looked at the role of mass media, behavioural interventions and biomedical interventions.

A review of observational studies found that antireteroviral therapy (ART) use in an HIV-infected member of an HIV-discordant couple is associated with lower risk of HIV transmission to the uninfected partner compared to untreated discordant couples (risk ratio (RR) 0.34; 95% CI 0.13, 0.92) [23]. Mass treatment after three rounds of treatment of all community members for STIs indicated no effect of the intervention with the risk ratio (RR) for HIV incidence being 0.97 (95% CI 0.81, 1.2) [24]. A combination of mass treatment and STI management trials showed a significant (12%) reduction in the prevalence of syphilis for those receiving a biomedical STI intervention (RR 0.88, 95% CI 0.80, 0.96) [24]. These interventions also showed a significant 51% reduction in the prevalence of Gonorrhoea (RR 0.49, 95% CI 0.31, 0.77), while no improvement was observed in Chlamydia (RR 1.03, 95% CI 0.77, 1.4) [24]. These findings suggest that effective interventions can prevent the spread of infection, if undertaken in reproductive age. Mass treatments and behavioural interventions to increase awareness can significantly reduce the incidence of STI while biomedical STI interventions can reduce the incidence of gonorrhoea, and chlamydia.

#### Peri-conceptual folic acid supplementation for preventing neural tube defects (NTDs)

Use of folic acid three months before conception to three months after conception, is known to reduce the risk of a first occurrence and a recurrence of NTDs [25,26]. Folic acid deficiency has been linked to adverse outcomes of pregnancy like LBW, antepartum haemorrhage and perinatal mortality [27]. It is recommended that all women of childbearing age should consume 0.4 mg of folic acid per day for the purpose of reducing the risk of a pregnancy with NTDs [28]. During pregnancy, the requirement may go up to at least 600 mcg, or even more up to 1000mcg.

Studies suggest that the use of folic acid is highly effective for the prevention of recurrent NTDs (RR 0.30; 95% CI: 0.14, 0.65) [29]. A Cochrane review also reported a protective effect of daily folic acid supplementation (alone or in combination with other vitamins and minerals) in preventing NTDs compared with no interventions/placebo or vitamins and minerals without folic acid (RR 0.28, 95% CI 0.15, 0.52) [30]. It is also shown to have some non-significant impact on the reduction of miscarriage (RR 1.10; 95% CI: 0.97, 1.26) [30] and stillbirth (RR 0.96; 95% CI: 0.51, 1.83) [30]. A meta-analysis of peri-conceptual folic acid fortification studies found reduction in primary incidence of NTDs by 41% (RR 0.59; 95% CI 0.52, 0.68] [31]. Findings suggest that periconceptional folic acid have a significant protective effect on NTDs, particularly in women who had a previous pregnancy affected by it (recurrent NTDs). New methods for the provision of folic acid are underway which includes the addition of folic acid in oral contraceptives. All women with a history of baby with NTDs should be advised and given folate to prevent its recurrence. Evidence from Cochrane review is weak regarding the impact of folic acid supplementation on stillbirths and miscarriages.

### Pregnancy interventions

#### Antenatal care visit model

The effectiveness of the content, frequency, and timing of visits in currently recommended programs for routine antenatal care is yet to be ascertained. Data from observational studies have shown that groups having more antenatal-care visits have lower maternal, fetal, and neonatal morbidity and mortality than those who have fewer antenatal-care visits [32]. Earlier review found no association of perinatal mortality with reduced visits compared to standard care (OR 1.06; 95% CI: 0.82, 1.36) [33]. However, a recent Cochrane review found that perinatal mortality was increased for those randomised to reduced visits rather than standard care (RR 1.14; 95% CI: 1.00, 1.31). In the subgroup analysis, for high-income countries (HICs) the association was non-significant (RR 0.90; 95% CI 0.45, 1.80); but for LMICs perinatal mortality was significantly higher in the reduced visits group (RR 1.15; 95% CI 1.01, 1.32) [34]. Perception of mothers for the quality of prenatal care was not affected by the number of visits (RR 1.0; 95% CI 0.98, 1.01) [34]. On the other hand, no difference in the rate of preterm birth (RR 0.87; 95% CI 0.47, 1.60) and LBW (less than 2500 g)(RR 1.03; 95% CI 0.73, 1.46) were found between women who received group antenatal care compared with women receiving standard one-to-one care [35]. The number of antenatal care visits should be based of how effectively interventions can be delivered in a timely way during pregnancy because the purpose of routine antenatal care is to deliver effective and appropriate screening, preventive, or treatment interventions.

#### Iron and folic acid supplementation during pregnancy for maternal anaemia

During pregnancy, mothers who have iron deficiency anaemia have shown to have inadequate weight gain needed to maintain a healthy fetus; have weak immune system causing them to be more prone to infections; have heavy placentas; and are at a higher risk of neonates being born either prematurely or with LBW [36-39]. It is suggested that the high iron demand during pregnancy cannot be fulfilled from diet alone, so iron supplementation is vital in these months. Pregnant women are routinely provided with iron supplementation throughout pregnancy. Along with this, other interventions are now being employed in order to reduce iron deficiency and these include iron fortification, health and nutritional education and the control of parasitic infections along with increasing sanitation standards [40].

Maternal outcomes are shown to be greatly improved with iron supplementation. Women taking iron supplements were less likely to have LBW newborns (below 2500 g) compared to controls (RR 0.81; 95% CI 0.68, 0.97) with greater mean birth weight (mean difference (MD) 30.81g; 95% CI 5.94, 55.68) [41]. Preventive iron supplementation reduces the risk of maternal anaemia at term by 70% (RR 0.30; 95% CI 0.19, 0.46) and iron deficiency at term by 57% (RR 0.43; 95% CI 0.27, 0.66) [41]. Moderately significant evidence was found by Yakoob et al. stating that daily iron supplementation resulted in 73% reduction in the incidence of anaemia at term (RR 0.27; 95% CI: 0.17, 0.42) and 67% reduction in iron deficiency anaemia at term (RR 0.33; 95% CI: 0.16, 0.69) compared to no intervention/placebo [42]. A review on folic acid supplementation during pregnancy showed no conclusive evidence for the impact on pregnancy outcomes such as preterm birth (RR 1.01; 95% CI 0.73, 1.38), and stillbirths/neonatal deaths (RR 1.33, 95% CI 0.96, 1.85). However, improvements were seen in the mean birth weight (MD 135.75g, 95% CI 47.85, 223.68) and reduction in the incidence of megaloblasticanaemia (RR 0.21, 95% CI 0.11, 0.38) [43]. Exiting evidence suggests that providing mothers with iron supplementation during pregnancy reduces the risk of being anaemic near or at term. Although periconceptional folate supplementation has a clear protective benefit against occurrence of NTDs, antenatal folate supplementation in combination with iron or multivitamins has a limited role in prevention of perinatal mortality or morbidity. There are relatively few studies in this regard and a stronger recommendation can be made when results of more studies will be available.

#### Tetanus immunisation in pregnancy for preventing neonatal tetanus

Neonatal tetanus is a form of generalised tetanus that has a high fatality rate. It usually occurs through infection of the unhealed umbilical stump, particularly when the stump is cut with an unsterile instrument [44]. Neonatal tetanus presents mostly (90%) during the first 3-14 days of life with the majority presenting at 6-8 days. The clinical presentation is very distressing and the case fatality rates tend to be very high even if full intensive care facilities are available. Therefore preventive measures are more effective than treatment options [45,46].

A Cochrane review by Dimecheli 2013, found 2 very old trials (one on non-pregnant women). One trial compared the effectiveness of tetanus toxoid with influenza vaccine and reported a non-significant reduction in neonatal deaths (RR 0.57; 95% CI: 0.26, 1.24) and 43% vaccine effectiveness [47]. Another trial assessed the effectiveness of tetanus-diphtheria toxoid in comparison with cholera toxoid in preventing neonatal mortality by 80% (RR 0.20; 95% CI 0.10, 0.40) with vaccine effectiveness as 80% [47].However, Blencowe 2010, added one well controlled cohort with one controlled trial and found a significant reduction in neonatal tetanus mortality by 94% (RR 0.06; 95% CI 0.02, 0.20) [48]. Despite the available low cost immunisation and feasibility of reaching high coverage even in weak healthcare systems, the expected reduction in tetanus neonatal mortality has not been achieved yet. It is expected that with recent investments in the campaign for maternal and neonatal tetanus elimination, there will be substantial progress in reduction of mortality due to this illness.

#### Prevention and management of malaria in pregnancy

Prevention of malaria in pregnancy is especially important because most of these infections remain asymptomatic, and therefore remain undetected and untreated [49]. There is a three-pronged approach to control malaria in pregnancy as suggested by WHO: it includes the use of insecticide-treated nets (ITNs) and antimalarial drugs, either through intermittent preventive therapy (IPT) or case management [50].

i) Prophylactic antimalarial for prevention of malaria in pregnancy

For malaria prophylaxis, IPTp with sulfadoxine-pyrimethamine (SP) is recommended for all pregnant women at each scheduled antenatal care visit. WHO recommends a schedule of four antenatal care visits.

- The first IPTp-SP dose should be administered as early as possible during the 2^nd^trimester of gestation

- Each SP dose should be given at least 1 month apart

- The last dose of IPTp with SP can be administered up to the time of delivery, without safety concerns

An unpublished meta-analysis by Kayentao et al. examined the number of IPTp doses that need to be administered during pregnancy to achieve the maximal beneficial effects of IPTp. The meta-analysis claimed that 3 or more doses (median of 4 doses) of IPTp with SP was superior to the standard 2 dose regimen in preventing LBW rates (reduction of 21% (95% CI 8, 32%) both in HIV infected and uninfected pregnant women and in all gravidity groups. Furthermore, women who received a median of 4 doses of IPTp-SP compared to those on the 2-dose regimen also had a lower risk of moderate-severe maternal anaemia, maternal malaria at delivery, and placental malaria. The meta-analysis, which included two trials in areas of Burkina Faso and Mali where the efficacy of SP remains high, showed that even in areas of high SP efficacy, 3 doses of SP were more effective than two doses [51]. Antimalarial drugs did not show improvements in reducing maternal deaths (RR 0.34; 95% CI: 0.04, 3.27) or perinatal deaths (RR 1.02; 95% CI: 0.73, 1.43) [52]. Routine antimalarial drugs reduce antenatal parasitaemia when given to pregnant women in malaria endemic areas. Greater benefit is seen in primi- and secundi-gravidas. SP regimen is feasible, though their regular use may precipitate increased risk of resistance. It is also associated with lower incidence of LBW (35%) and all-cause child mortality (18%). Two-dose IPT with SP when compared with placebo in women during their first or second pregnancy was found to reduce placental malaria (RR 0.48; 95% CI: 0.35, 0.68), LBW (RR 0.71; 95% CI: 0.55, 0.92), and anaemia (RR, 0.90; 95% CI: 0.81, 0.99) [53].

ii) Insecticide treated bed nets for preventing malaria in pregnancy

An insecticide-treated net (ITN) is a mosquito net that repel/disables and/or kills mosquitoes coming into contact with insecticide on the netting material. All mosquito nets act as a physical barrier, preventing access by vector mosquitoes and thus providing personal protection against malaria to the individual(s) using the nets. By reducing the vector population, ITNs, when used by a majority of the target population, provide protection for all people in the community, including those who do not themselves sleep under net. There are two categories of ITNs: conventionally treated nets and long-lasting insecticidal nets.

Langeler etal.conducted a review of five trials which showed that ITNs provided 17% protective efficacy (PE) compared to no nets (RR 0.83, 95% CI 0.76, 0.90), and 23% PE compared to untreated nets (RR 0.77, 95% CI 0.63, 0.95). About 5.5 lives (95% CI 3.39, 7.67) can be saved each year for every 1000 children protected with ITNs [54]. Results by Gamble at al. show that ITNs, compared with no nets, reduced placental malaria in all pregnancies (RR 0.79, 95% CI 0.63, 0.98). They also reduced LBW (RR 0.77, 95% CI 0.61, 0.98) and fetal loss in the first to fourth pregnancy (RR 0.67, 95% CI 0.47, 0.97), but not in women with more than four previous pregnancies. It reduces peripheral and placental parasitaemia by 23% (RR 0.77; 95% CI 0.66, 0.90) [55,56], increases mean birth weight by 55g (95% CI 21, 88) [55,56], and decreases the risk of fetal loss in the by 33% (RR 0.67; 95% CI 0.47, 0.97) [55,56]. Eisele et al. estimated that malaria prevention interventions in pregnancy (IPTp and ITNs) have a pooled PE of 35% (95% CI: 23-45%) for reducing the prevalence of LBW in the first or second pregnancy in areas of stable P. falciparum transmission [57]. ITNs, therefore, should be an integral part of strategies to prevent malaria in pregnant women living especially in areas where malaria is endemic.

#### Smoking cessation in pregnancy for improving birth outcomes

Smoking during pregnancy has serious consequences on the health and wellbeing of the newborn child. It has important deleterious effects on the baby at birth and throughout the early development of the child. The smoking cessation strategies in pregnancy include provision of advice and counselling using various tools (written and electronic resources and telephone support), cognitive behavioural therapy (CBT) and motivational interviewing, advice and counselling based on feedback of fetal health status or measurement of by products of tobacco smoking in the mother, provision of pharmacological agents such as nicotine replacement therapy and bupropion, social support and encouragement, including the use of rewards for cessation and other interventions such as hypnosis.

Smoking cessation programs in pregnancy have shown to reduce smoking in late pregnancy (RR 0.94, 95% CI: 0.93 to 0.96) [58]. This also has an impact on reducing LBW (RR 0.83, 95% CI 0.73 to 0.95) and preterm birth (RR 0.86, 95% CI 0.74 to 0.98), with a 53.91g (95% CI 10.44, 95.38 ) increase in mean birth weight [58]. However, the difference in smoking cessation in later pregnancy after using nicotine replacement therapy was not significant when compared with placebo group (RR 1.33, 95% CI 0.93, 1.91) [59]. It is important that smoking cessation programs be implemented in all maternity care settings. Smoking cessation and relapse prevention strategies should be routine parts of antenatal care as, for example, the measurement of blood pressure. Counselling and convincing patients for smoking cessation is a difficult job and it should be emphasized that midwives, general practitioners, and obstetricians support population-wide strategies for smoking control in the whole community to reduce/prevent/stop smoking by young people.

#### Prevention and management of syphilis

Maternal syphilis is related with adverse outcome in newborns. In 2008, the WHO estimated that 1.86 million cases of syphilis occur globally among pregnant women each year and that a large proportion of them are untreated or inadequately treated. Up to one third of the women attending antenatal care (ANC) clinics are not tested for syphilis [60].

Treatment of syphilis is associated with a reduction in preterm delivery (RR 0.36; 95% CI: 0.27, 0.47) [61]. Observational studies have shown a reduction in stillbirths and neonatal mortality (RR 0.18; 95% CI: 0.10, 0.33 and RR 0.20; 95% CI: 0.13, 0.32 respectively) [61]. Review by Walker at al.did not find studies that met predetermined criteria for comparison, however, they did conclude that penicillin are effective in treating syphilis but more studies are needed to establish the most effective dosage and duration of therapy [62]. According to the CDC guidelines, the treatment of syphilis during pregnancy includes penicillin and the doses depend upon the stage of syphilis [63]. For primary, secondary, and early latent syphilis the recommendation is to give benzathine penicillin-G 2.4 million units intramuscular in one dose. For females with late latent syphilis or latent syphilis with unknown duration the recommendation is for 7.2 million units of benzathine penicillin G administered as three doses (2.4 million units per dose) given intramuscularly for three weeks. The review on antibiotics given for the management of syphilis did not have any trial that met the eligibility criteria. However, observational studies showed benefits in lowering the incidence of neonatal mortality and preterm delivery.

#### Prevention and management of HIV and prevention of mother to child transmission (PMTCT) in pregnancy

Antiretroviral (ARV) drugs reduce viral replication and can reduce mother-to-child transmission of HIV either by lowering plasma viral load in pregnant women or through post-exposure prophylaxis in their newborns. In rich countries, highly active antiretroviral therapy (HAART) which usually comprises three drugs has reduced the mother-to-child transmission rates to around 1-2%. New evidence published in Lancet Infectious Diseases based on the Kesho Bora Studies state the giving a combination of three ARV drugs to pregnant mothers with HIV infection fromthelasttrimester, through delivery and six months of breastfeedingreducestheriskof transmitting HIV to the baby and improves survival. Infants of mothers whose virus is fully suppressed (undetectable) by triple-ARVs at the time of delivery have a very low risk of HIV infection (only 2.7% by the age of one year). It is therefore important to start ARVs early in pregnancy, ideally before pregnancy, for all women who require antiretroviral treatment (ART) (CD4 count at or below 350 cells/mm^3^). Giving HIV-positive pregnant women and that planning pregnancy priority access to HIV testing, CD4 count and ARVs will help eliminate mother-to-child transmission of HIV [64].

Vitamin A supplementation was found to reduce the risk of HIV infection in child (RR 1.04; 95% CI: 0.87, 1.24) [65] thus being useful for PMTCT. Vaginal disinfection, on the other hand, did not find any impact on reducing the transmission of HIV to child (RR 0.93; 95%CI 0.63, 1.38) [66].

A new study led by the WHO's Department of Reproductive Health and Research shows that if HIV-positive pregnant women are given a combination of antiretroviral (ARV) drugs from late in pregnancy until six months into breastfeeding, rather than a short course of drugs that ends at delivery, the likelihood of their babies becoming infected with HIV are over 40% lesser than with no such treatment [67].

The Kesho Bora study shows that a significant reduction in infant infection can be achieved when pregnant women with a CD4 immune cell count of 200-500 cells/ mm3 are given a combination of three ARVs to prevent transmission. This treatment should start in their last trimester of pregnancy, continuing through birth and six months of breastfeeding. This was shown to reduce the risk of transmitting HIV to the baby and improved survival compared with babies of mothers with HIV who are given the current WHO-recommended short-course ARV regimen in late pregnancy and around the time of delivery. An infant of mothers whose virus is fully suppressed undetectable by triple-ARVs at the time of delivery has a very low risk of HIV infection (only 2.7% by the age of one year). It is therefore important to start ARVs early in pregnancy, ideally before pregnancy, for all women who require antiretroviral treatment (ART) (CD4 count at or below 350 cells/mm3). Giving HIV-positive pregnant women and those planning pregnancy priority access to HIV testing, CD4 countand ARVs will help eliminate mother-to-child transmission of HIV [67].

The review by Read et al. identified 6 RCTs, of which, one RCT was on the efficacy of elective caesarean section (ECS) for prevention of MTCT of HIV-1. Among HIV-1-infected women not taking antiretroviral (ARVs) during pregnancy or taking only zidovudine, ECS was found to be efficacious for prevention of MTCT of HIV-1 [68].

#### Calcium for hypertension in pregnancy

Hypertension, with or without proteinuria, is an important cause of maternal and perinatal mortality and morbidity globally [69,70]. It complicates pregnancy and is responsible for maternal deaths [71]. Therefore, strategies to reduce the risk of these hypertensive disorders should be given high priority to save thousands of lives of mothers, especially in developing countries.Based on observations of communities in Guatemala and Ethiopia, it has been seen that women with a high calcium intake in their diet have a lower prevalence of pre-eclampsia and eclampsia [72]. This was further supported by other epidemiological and clinical studies [72-75]. A possible mode of action of calcium could be that it reduces parathyroid hormone release and intracellular calcium, and so decreases smooth muscle contractility and, therefore, vasoconstriction [76,77]. Calcium can also act directly on uterine smooth muscle to reduce contractility and prevent pre-term labour and delivery [76,77].

Calcium supplementation can be an attractive, potential intervention to reduce the risk of a woman developing pre-eclampsia.

All studies showed that calcium supplementation has an effect on the reduction of pre-eclampsia (RR 0.45; 95% CI: 0.31, 0.65) [78].Hofmeyer 2007 also showed that high blood pressure was reduced with calcium supplementation when compared with placebo (RR 0.65, 95% CI 0.53, 0.81), as was pre-eclampsia (RR 0.48; 95% CI 0.33, 0.69) [79]. The effect was greatest for women at high risk (RR 0.22; 95% CI 0.12, 0.42) and for those with low baseline calcium intake (RR 0.36; 95% CI 0.18, 0.70) [79,80]. The composite outcome maternal death or serious morbidity was reduced (RR 0.80; 95% CI 0.65, 0.97). There was no overall effect on the risk of preterm birth or stillbirth or death before discharge from hospital [79,80]. Jabeen et al. showed borderline reduction in stillbirths (RR 0.81, 95 % CI 0.63, 1.03)with calcium supplementation [81].

Calcium supplementation significantly reduced the risk of pre-eclampsia by 55% and by 64% in women with low dietary intake of calcium. It also had a significant protective effect on maternal mortality and serious morbidity and preterm birth. Its effect on, LBW or stillbirth was, however, non-significant. More large-scale trials need to be conducted to assess its safety profile before it can be routinely introduced into clinical practice.

#### Anti-platelet agents (low dose aspirin) for prevention of pre-eclampsia

Pre-eclampsia also leads to deficiency of prostacyclin, a vasodilator, and produces excessive amounts of thromboxane, which acts by stimulating platelet aggregation and causing platelet derived vasoconstriction [82]. It is due to these observations that it is said the anti-platelet agents, especially low dose aspirin, can prevent or delay the development of pre-eclampsia.

The results show that that when anti-platelet agents are given during pregnancy, it leads to consistent reduction in the risk of preterm births (<34 weeks) (RR 0.92, 95%CI 0.88, 0.97), pre-eclampsia (RR 0.83, 95% CI 0.77, 0.89) and other adverse effect of pregnancy [83]. Importantly for the fetus there was a significant reduction of 17% in all types of deaths (fetal, neonatal, infant) (RR 0.86, 95%CI 0.76, 0.98) [83]. It is also noted that anti-platelet agents given to pregnant mothers who had gestational hypertension benefit even more with a significant reduction of 40% in the incidence of pre-eclampsia (RR 0.60 95%CI 0.45, 0.78) [83]. On the other hand, a review Jabeen et al. did not show significant impact on reducing the risk of stillbirths (RR 1.15; 95% CI: 0.88, 1.49) [81]. Another review also reported reduce incidence of pre-eclampsia with anti-platelet agents (RR 0.90, 95% CI 0.84, 0.97) [84].

We would recommend that anti-platelet agents should be given to pregnant women at high risk of pre-eclampsia or those with gestational hypertension, since they lead to reduction of vast adverse outcomes of pregnancy both for the mother and newborn.

#### Anti-hypertensive for treating severe hypertension

Hypertensive disorders of pregnancy, as a group, are a major cause of maternal and perinatal morbidity and mortality in both developing and the developed world [85,86]. The International Society for the Study of Hypertension in Pregnancy defines hypertension as a diastolic blood pressure of 90 mmHg or above on two consecutive occasions at least four hours apart, or a single diastolic blood pressure of 110mmHg or more [87].

Women allocated calcium channel blockers rather than hydralazine were less likely to have persistent high blood (RR 0.37, 95% CI 0.21, 0.66) [88]. Ketanserin was found to be associated with more persistent high blood pressure than hydralazine (RR 4.79, 95% CI 1.95, 11.73), but fewer side-effects (RR 0.32, 95% CI 0.19, 0.53). Both nimodipine and magnesium sulphate were associated witha high incidence of persistent high blood pressure, but a single study showed that this risk was lower for nimodipine compared to magnesium sulphate (RR 0.84, 95% CI 0.76 to 0.93) [88]. Oral beta-blockers decrease the risk of severe hypertension (RR 0.37, 95% CI 0.26 to 0.53) and the need for additional antihypertensive (RR 0.44, 95% CI 0.31 to 0.62) [89].

The rationale for giving anti-hypertensive is that they will be dilatory in progression to severe disease with drastic complications for the mother and her baby. They may also reduce the risk of preterm delivery and placental abruption and improve fetal growth. Until better evidence is available, the choice of antihypertensive should depend on the clinician’s experience and familiarity with a particular drug and on what is known about adverse effects. Exceptions are diazoxide, ketanserin, nimodipine and magnesium sulphate, which are probably best avoided. The use of antihypertensive for treating severe hypertension in pregnant women is likely to bring an improvement in the neonatal and maternal outcomes.

#### Management of pregnancy-induced hypertension (PIH)/eclampsia

Pre-eclampsia is defined as a disorder during pregnancy with raised blood pressure and proteinuria, which may involve multiple systems including liver, kidneys, clotting system and brain. If it is complicated by convulsions (fits), then the condition is called eclampsia. Anticonvulsants like magnesium sulphate are used for eclamptic seizures and were introduced for women with pre-eclampsia in the belief that they would prevent the onset of seizures. This profile compares magnesium sulphate with other agents like diazepam, phenytoin and lytic cocktail for control of eclampsia; and also reviews the pros and cons of using it in women with pre-eclampsia as a prophylaxis vs. no anticonvulsants.

Magnesium sulphate therapy usually starts with an intravenous loading dose usually 4g but some advocate 6g, with therapy being continued either for 24 hours in total or until 12 to 24 hours after delivery. This is followed by either intramuscular (IM) or intravenous (IV) maintenance therapy. The IM maintenance regimen is usually 10 g given with the loading dose, and then 5 g every four hours. The IV infusion is usually 1 g/hour, although 2 g/hour is used by some.

Magnesium sulphate for pregnancies with pre-eclampsia had a significant impact on reducing stillbirths (RR 0.99; 95% CI: 0.87, 1.12) when compared with placebo/no treatment [81]. When comparing magnesium sulphate to diazepam, magnesium sulphate was associated with a reduction in maternal death (RR 0.59, 95% CI 0.38, 0.92) and recurrence of seizures (RR 0.43, 95% CI 0.33, 0.55) [90]. When compared with phenytoin, magnesium sulphate was associated with a substantial reduction in the recurrence of seizures (RR 0.34, 95% CI 0.24, 0.49). The trend in maternal mortality favours magnesium sulphate, but the difference does not reach statistical significance (RR 0.50, 95% CI 0.24, 1.05) [91]. On the other hand, when magnesium sulphate was compared with placebo or no anticonvulsant, it led to halved the risk of eclampsia (RR 0.41, 95% CI 0.29, 0.58), with a non-significant reduction in maternal death (RR 0.54, 95% CI 0.26, 1.10) but no clear difference in serious maternal morbidity (RR 1.08, 95% CI 0.89, 1.32) [92]. Magnesium sulphate was also compared with lytic cocktail and it was associated with fewer maternal deaths (RR 0.14, 95% CI 0.03, 0.59) and was better at preventing further seizures (RR 0.06, 95% CI 0.03, 0.12) than lytic cocktail [93].

Magnesium sulphate is an inexpensive drug and can be conveniently used in low income countries. Our review indicates that this drug significantly reduces the progress to eclampsia when given to women with pre-eclampsia compared to placebo. It should, therefore, be considered for those pre-eclamptic cases where there is a concern about the development of eclampsia. Clinical monitoring is necessary and can be achieved if trained staff is available. Serum monitoring is not necessary. For eclampsia, magnesium sulphate is now preferred over diazepam, phenytoin or lytic cocktail. It is significantly better in preventing maternal mortality and recurrence of seizures.

#### External cephalic version for breech presentation at term (>36 weeks)

Malpresentation places a healthy fetus and mother at increased risk of a complicated vaginal birth or caesarean section. It is not surprising that, over the years, the possibility of turning the baby from the breech to the cephalic presentation (ECV) has intrigued some obstetric caregivers. Before the mid-1970s, ECV was usually attempted before term because of the belief that the procedure would seldom be successful at term. Subsequent studies showed that with the use of tocolysis, ECV could be achieved in a substantial proportion of women with breech presentation at term (37 completed weeks of pregnancy or more). Predictors of unsuccessful version include engaged presenting part, fetal head not easily palpable and tense uterus [94].

Tocolytic drugs, in particular beta stimulants, were effective in increasing cephalic presentations in labour by 38% (RR 1.38, 95% CI 1.03,1.85) and in reducing the number of caesarean sections by 18% (RR 0.82, 95% CI 0.71, 0.94) [95]. Initially, successful ECV at a late stage of pregnancy was considered to have become possible only because of the use of tocolytic drugs to relax the uterus. However, later studies showed that ECV at term was frequently possible without tocolysis. The overall success rate was 60% in a systematic review of RCTs where some trials included facilitation and others did not [96].

When postural management with pelvic elevation for breech presentation was compared with a control group reported the similar rates for non-cephalic births, caesarean section and Apgar scores below 7 at one minute, regardless of whether ECV was attempted or not(RR 0.98; 95% CI 0.84, 1.15; RR 1.10; 95% CI 0.89, 1.37; RR 0.88; 95% CI 0.50, 1.55 respectively)[97]. When comparing births with ECV for term births, no significant differences in the incidence of Apgar score ratings below seven at one minute (RR 0.95, 95% CI 0.47, 1.89) or five minutes (RR 0.76, 95% CI 0.32, 1.77), low umbilical artery pH levels (RR 0.65, 95% CI 0.17, 2.44), neonatal admission (RR 0.36, 95% CI 0.04, 3.24), perinatal death (RR 0.34, 95% CI 0.05, 2.12), nor time from enrolment to delivery (Weighted Mean Difference (WMD) -0.25 days, 95% CI -2.81 to 2.31) were found [98]. For term breech delivery, perinatal or neonatal death (excluding fatal anomalies) or serious neonatal morbidity was reduced with planned caesarean section (RR 0.33, 95% CI 0.19, 0.56). This reduction was less for countries with high national perinatal mortality rates [99]. Evidence exists in which moxibustion has no impact in reducing the need for ECV (RR 0.67 95% CI 0.34, 1.32) [100] but there is a need for well-designed RCTs to establish further evidence of the effectiveness of the intervention.

During an external cephalic version, practitioners use their hands on the woman’s abdomen to gently try to turn the baby from the breech position. There is sufficient evidence to conclude that external cephalic version at term reduces the chances of non-cephalic births and caesarean sections. There is lack of evidence from RCTs to assess the complication of cephalic version at term. Insufficient evidence exists to support the use of moxibustion to correct breech presentation.

#### Management of preterm, premature rupture of membranes

A) Management of premature rupture of membranes (PROM) (induction of labour)

Rupture of membranes before the onset of labour complicates 5-10% of all pregnancies, referred to as PROM (premature rupture of the membranes) [101]. A minimum of 60% of these cases occur in pregnant women at term. Infection can be cause or a consequence of PROM. One way to prevent infections after PROM at term would be to expedite delivery by inducing labour, in which case pre-labour antibiotics may not be indicated. But in situations where expectant management is preferred, antibiotics may be beneficial in terms of reducing the likelihood of infection in the mother and her baby. This review compiles the existing evidence on the benefits and risks of the use of antibiotics in PROM at or near term.

Early delivery increased the incidence of caesarean section (RR 1.51; 95% CI 1.08, 2.10). There was no difference in the overall perinatal mortality (RR 0.98, 95% CI 0.41, 2.36), intrauterine deaths (RR 0.26;95% CI 0.04, 1.52) or neonatal deaths (RR 1.59, 95% CI 0.61, 4.16) when comparing early delivery with expectant management [102].

There is insufficient evidence to guide clinical practice on the benefits and harms of immediate delivery compared with expectant management for women with PPROM.

B) Management of preterm, premature rupture of membranes (PPROM) (use of antibiotic)

Management of PROM includes administration of any antibiotic for women with PPROM, inculcating all beta-lactam antibiotics (including penicillin) and macrolides.

The use of antibiotics was associated with a statistically significant reduction in chorioamnionitis(RR 0.66, 95% CI 0.46, 0.60) [103], preterm birth (RR 0.79, 95% CI 0.71, 0.89) [103] and neonatal infections including pneumonia (RR 0.67, 95% CI 0.52, 0.85) [103], which would support their routine clinical use in cases of pPROM. It did not, however, significantly impact perinatal mortality and neonatal mortality. Cousenset al. included 18 RCTs from high-income countries and provided strong evidence that antibiotics for pPROM reduce the risk of respiratory distress syndrome (RR 0.88; 95% CI 0.80, 0.97), and early onset postnatal infection (RR 0.61; 95% CI 0.48, 0.77) [104]. The data was also found to be consistent with a reduction in neonatal mortality (RR 0.90; 95% CI 0.72, 1.12) [104].

Any antibiotics, administered as prophylaxis, by any route, to women at gestational age 36 weeks or beyond, with premature rupture of the membranes, can be used to manage this condition. There is not enough evidence to recommend routine use of antibiotics in women with PROM at term, although there was a 36% reduction in chorioamnionitis. Antibiotics may be reserved only for women that develop clinical indications of an infection, rather than prescribing it for all women in general. There were no clear neonatal advantages either. More trials are needed to evaluate their use, especially in women with PROM with expectant management or with a policy of delayed induction.

C) Corticosteroid use in preterm labour

One of the gravest outcomes of preterm labour is babies being born with respiratory distress syndrome (RDS), as this is the primary cause of early neonatal death and disability [105]. RDS occurs as a complication of preterm delivery and it is present in about 20% of LBW babies (<2500g) and about 66% of extremely LBW babies (<1500g) [105]. The reason for the development of RDS is a combination of poor lung development, immaturity of organs and a deficiency of surfactant. The role of corticosteroids when given prior to preterm birth shows that they can decrease the incidence of RDS and neonatal mortality in babies [106]. This occurs from the theory that steroids can cause premature liberation of surfactant by alveoli and the surfactant produced will help in preventing the alveoli from collapsing. In the absence of surfactant the alveoli collapse during expiration and the following inspiration needs more effort to open the alveoli. If there is no surfactant this cycle of the alveoli collapsing continues and finally leads to fatigue, decreased respiratory effort, hypoxia, cyanosis, acidosis, and eventually death [107].

Antenatal steroids decrease neonatal mortality among preterm infants (<36 weeks gestation) by 31% (RR 0.69; 95% CI 0.58, 0.81). RCTs from middle-income countries suggests 53% mortality reduction (RR 0.47; 95% CI 0.35, 0.64) and 37% morbidity reduction (RR 0.63; 95% CI 0.49, 0.81) [108]. Treatment with corticosteroids showed a statistically significant reduction of 23% in combined incidence of fetal and neonatal deaths (RR 0.77, 95%CI 0.67, 0.89), and this result was mainly due to the reduction seen in neonatal deaths (RR 0.69, 95% CI 0.58, 0.81). Antenatal corticosteroids were tested for the reduction ofmaternal death but the result failed to reach statistical significance (RR 0.98, 95% CI 0.06, 15.50) [105].There was also a significant reduction in the incidence of RDS (RR 0.66, 95% CI 0.59,0.73),moderate to severe RDS(RR 0.55, 95% CI0.43, 0.71),cerebroventricular haemorrhage (CVH) and severe CVH (RR 0.54, 95% CI 0.43, 0.69) [105]. A Cocharane review of 10 trials showed that dexamethasone decreased the incidence of intraventricular haemorrhage compared with betamethasone (RR 0.44, 95%CI 0.21, 0.92) [109].

#### Management of unintended pregnancy

Numbers of unsafe abortions have increased from 19.7million in 2003 although the overall unsafe abortion rate remains unchanged at about 14 unsafe abortions per 1000 women aged 15–44 years [14]. WHO defines these abortions as procedures that women undergo in order to terminate an unwanted pregnancy either in surroundings that are far below the medical standards or done by unskilled individuals, or both [110]. The reasons for high rates of unsafe abortions in the developing world are that in some countries abortions are considered illegal or legal abortions are not offered, forcing females with unplanned pregnancy to either obtain secret abortions by untrained medical workers or self-induce abortions leading to the risk of complications. Females who undergo unsafe abortions are at the risk of developing complications which in most cases lead to long hospital stay and can also lead to mortality. It is stated that the commonest causes of mortality in females undergoing unsafe abortions are haemorrhage, infections and, in some cases, poisons that were used in self-inducing abortions [111]. It is reason like these that makes it vital for clinics and hospitals, especially those in the developing countries, to have proper post-abortion care for patients. The fundamentals of post-abortion care should include having a medical staff skilled enough to handle all types of complications, along with having facilities for uterine evacuation procedures, along with referral of all patients to advanced sexual and reproductive health services. Lastly, females should be provided with counselling on contraceptives, family planning and told about the risks of unsafe abortions [112].

Another option to decrease the number of unsafe abortions and its complications would be by legalizing abortions, along with promoting the use of contraceptives or providing females proper counselling on these topics. It is seen that in those countries where abortion is legalized, mortality has considerably declined. This notion would be highly recommended for all developing nations where abortion is still not legalized, especially countries where religious prohibition and social norms do not allow this to occur.

According to the WHO guidelines on safe abortion 2003 [113], abortion services should be provided by well-trained health personnel to pregnant women who elect for the procedure. It must be supported by policies, regulations and a health systems infrastructure, including equipment and supplies, so that women can have rapid access to these services.

## Cross cutting interventions

### Home visits for women and children across the continuum of care

It is difficult to provide expert medical care to each and every person at community level especially in developing countries. To support the basic primary health care infrastructure, different community based intervention packages have been introduced to train persons in the society who has some background medical knowledge e.g. community health workers. These individuals with proper training, can function as community activists, opinion leaders, or health promoters, and can share their knowledge with community members, including pregnant women and their families. However as the background of each community health worker is different, a particular integrated approach could not be developed with public sector programs to target a particular goal. Different community based intervention packages have been developed to train community health workers for specific tasks like antenatal and postnatal care etc. [114,115].

Improved perinatal care practices led to a drop in maternal mortality rate (OR 0.62; 95% CI 0.39, 0.98) [116]. Home based Neonatal care packages led to a decrease in perinatal mortality (RR 0.71; 95% CI 0.61, 0.84) [114]; as well as incidence of stillbirths (RR 0.87; 95% CI 0.73, 1.03) [115]. Another review showed a reduction in neonatal mortality (RR 0.76; 95% CI 0.68, 0.84) as well as incidence of stillbirths (RR 0.84; 95% CI 0.74, 0.97) and perinatal mortality (RR 0.80; 95% CI 0.71, 0.91) [117]. The intervention packages included home visits during the neonatal period, home-based treatment for illness and community mobilization efforts showed a reduced risk of neonatal death (RR: 0.62; 95% CI 0.44, 0.87) and stillbirth (RR: 0.76; 95% CI 0.65, 0.89) [118].

The available data suggests that introduction of community based intervention packages can improve maternal and neonate survival. The results from Cochrane review are very promising and showed impact on reducing stillbirths, perinatal mortality and neonatal mortality.

## Discussion

The review aimed to document the essential pre-pregnancy and pregnancy interventions for improved maternal, newborn and child health. It covers pre-pregnancy interventions including family planning, prevention and management of STI and HIV and folic acid suuplementation prior to pregnancy amongst other interventions. In order to achieve maximum impact of pre-pregnancy interventions, these interventions should be appropriately timed [119]. With regards to antenatal care, calcium and magnesium supplementation for prevention and management of pre-eclampsia and prophylactic and therapeutic uterotonics for prevention of postpartum heamorrahge are a couple of effective interventions that have the potential to save many maternal lives and prevent poor pregnancy outcomes. The implementation of the interventions reviewed in this paper will provide to be of paramount importance to improving the maternal and neonatal statistics especially in the developing countries, where there is a lot of potential work to be done. It is vital to understand that these interventions are central for LMIC where maternal and child health indicators are still not up to the standards and many lives are either lost or their quality compromised owing to a dearth of provision of essential interventions [120].

This review was designed to serve as a comprehensive and thorough document of essential pre-pregnancy and pregnancy interventions which could be used by both clinicians and policy makers. The review included all the recent Cochrane and non Cochrane reviews on specific interventions and the exercise included all essential pre-pregnancy and pregnancy interventions for maternal, newborn and child health. The data was summarized in a logical manner that can be utilized for further implementation and policy making to enable improvement in current health care system of LMICs. However, at times the quality of all included randomized controlled trials could not be ensured which limited the quality of the data obtained. Some reviews that were included were not updated recently and the data extracted from them might reflect facts that are not up to date. However, those reviews which had been updated by the authors were revised and the data extracted from within them was made up to date.

As stated earlier, the review is meant to be useful for health care practitioners as well as policy makers so that they may integrate and plan these interventions not only during the course of the pregnancy but also before pregnancy. It is essential to make policies and ensure that these essential interventions are delivered in a timely and effective manner.The integration of these interventions in the existing health services can not only improve outcomes presently but also lead to a better future of health care settings for both mothers and neonates. Long term sustainment of these improvements are necessary, therefore, it is essential to evaluate the impact of these interventions once streamlined into existing packages and programs. The transition to improved health care system must be made soon as it is becoming increasingly important to deliver these interventions at a large scale. With the deadlines of MDGs approaching upon us, immediate action is required to discuss these issues at policy making levels as well, so that we can find a way forward. Unless these problems are actively pursed and the suggestions given a thought, the issues related to maternal and newborn health care will keep on lingering upon us. Therefore, the review focuses on the approach which is critical to achieving the MDGs 4 and 5 – ensuring that we not only save lives to also address overall health and well-being.

## Conclusion

Needless to say,the global cost for maternal disability is very high, therefore, ensuring the prompt and effective delivery of these interventions will be an effective strategy at moving towards the achievement of the millennium development and it would also lead to a much needed decrease in the burden of disease related to chronic complications of pregnancy and childbirth. Quite promisingly, a decline of 47% is seen in the statistics related to maternal mortality since the 1990s around the globe. Most encouraging is the fact that since 1990, some countries in Asia and Northern Africa have more than halved the numbers related to maternal mortality. These advancements herald atime when the achievement of MDGS 4 and 5 is closer than it has ever been before. With the proper implementation of a set of essential interventions recommended in this paper, we can hope to see the promising status of both improved and sustained maternal, neonatal and child helath outcomes.The integration of the basic care recommended and discussed in this paper with the global healthcare settings will be an effective strategy towards attaining a better health care setting for mothers and their children.With each passing day, we must aim to build a future in which each mother and child receives the best of health care regardless of their financial status or the area in which they reside. This could only be possible if there is a global knowledge and implementation of thelife saving interventions mentioned in this review.

## Competing interests

We do not have any financial or non-financial competing interests for this review.

## Peer review

The reviewer reports for this article can be found in Additional File 1.

## Supplementary Material

Additional file 1 Peer review reports. Click here for file

## Declarations

This article has been published as part of *Reproductive Health* Volume 11 Supplement 1, 2014: Essential intervention for maternal, newborn and child health. The full contents of the supplement are available online at http://www.reproductive-health-journal.com/supplements/11/S1. Publication charges for this collection were funded by the Partnership for Maternal, Newborn & Child Health (PMNCH).
