# Supplementary material for: Essential pre-pregnancy and pregnancy interventions for improved maternal, newborn and child health
Source: Reprod Health. 2014 Aug 21;11(Suppl 1):S2. doi: 10.1186/1742-4755-11-S1-S2 (PMC4145858; doi:10.1186/1742-4755-11-S1-S2)
Supplement: Additional file 1 — Peer review reports. [file 1742-4755-11-S1-S2-S1.pdf]

Referee's comments to the authors– this sheet WILL be seen by the author(s) and published with the article

Title	**Essential Pre-pregnancy and pregnancy interventions for improved maternal, newborn and child health**
Author(s)	Zohra S Lassi, TarabMansoor, Rehana A Salam, Jai K Das, Zulfiqar A Bhutta
Referee's name	Adenike Grange

When assessing the work, please consider the following points, where applicable:

1. Is the question posed by the authors new and well defined? The question is well defined in the introduction paragraph.
2. Are the methods appropriate and well described, and are sufficient details provided to replicate the work? The "Methods" were omitted
3. Are the data sound and well controlled? By and large
4. Does the manuscript adhere to the relevant standards for reporting and data deposition? Fair
5. Are the discussion and conclusions well balanced and adequately supported by the data? Yes
6. Do the title and abstract accurately convey what has been found? Yes
7. Is the writing acceptable? Yes

Please make your report as constructive and detailed as possible in your comments so that authors have the opportunity to overcome any serious deficiencies that you find and please also divide your comments into the following categories:

- Major Compulsory Revisions (which the author must respond to before a decision on publication can be reached)
- Minor Essential Revisions (such as missing labels on figures, or the wrong use of a term, which the author can be trusted to correct)
- Discretionary Revisions (which are recommendations for improvement but which the author can choose to ignore)

Where possible please supply references to substantiate your comments.

When referring to the manuscript please provide specific page and paragraph citations where appropriate.

General comments:

Major compulsory revisions: Reduce length of Introduction.
Include Methodology
Include "Neonatal Resuscitation in the Essential package
Check on Efficacy of Withdrawal method versus Implant

Minor essential revisions: Syntax errors
Spelling errors
Punctuations
Word Spacing

eferee's comments to the authors– this sheet WILL be seen by the author(s) and published with the article

Title	**Essential Pre-pregnancy and pregnancy interventions for improved maternal, newborn and child health**
Author(s)	Zohra S Lassi, TarabMansoor, Rehana A Salam, Jai K Das, Zulfiqar A Bhutta
Referee's name	Marion Koso-Thomas

When assessing the work, please consider the following points, where applicable:

1. Is the question posed by the authors new and well defined?
2. Are the methods appropriate and well described, and are sufficient details provided to replicate the work?
3. Are the data sound and well controlled?
4. Does the manuscript adhere to the relevant standards for reporting and data deposition?
5. Are the discussion and conclusions well balanced and adequately supported by the data?
6. Do the title and abstract accurately convey what has been found?
7. Is the writing acceptable?

Please make your report as constructive and detailed as possible in your comments so that authors have the opportunity to overcome any serious deficiencies that you find and please also divide your comments into the following categories:

- Major Compulsory Revisions (which the author must respond to before a decision on publication can be reached)
- Minor Essential Revisions (such as missing labels on figures, or the wrong use of a term, which the author can be trusted to correct)
- Discretionary Revisions (which are recommendations for improvement but which the author can choose to ignore)

Where possible please supply references to substantiate your comments.

When referring to the manuscript please provide specific page and paragraph citations where appropriate.

General comments: Excellent review of the current maternal / infant situation and very informative

Major compulsory revisions: NONE

Minor essential revisions: some typographical errors

Discretionary revisions:

(continue on the next sheet)

Comments from the Supplement Editor

Please review the references carefully and ensure that the most appropriate and up-to-date references are cited. As an example, reference [94] is the Cochrane review on 2007 and there is an update review published in 2013.

As further examples, for the section on Calcium hypertension in pregnancy, appropriate references would be:

Based on observations of communities in Guatemala and Ethiopia, it has been seen that women with a high calcium intake in their diet have a lower prevalence of pre-eclampsia and eclampsia [83]. This was further supported by other epidemiological and clinical studies [84-87]. A possible mode of action of calcium could be that it reduces parathyroid hormone release and intracellular calcium, and so decreases smooth muscle contractility and, therefore, vasoconstriction[88,89]. Calcium can also act directly on uterine smooth muscle to reduce contractility and prevent pre-term labour and delivery [88, 89].

83. Belizán JM, Villar J**. The relationship between calcium intake and edema, proteinuria and hypertension gestosis. A hypothesis.** American Journal of Clinical Nutrition 1980; 33:2202-10.

84. Villar J, Belizan JM, Fischer PJ: Epidemiologic observations on the relationship between calcium intake and eclampsia. Int J Gynaecol Obstet 1983, 21(4):271-278.

85. Belizán JM, Villar J, Bergel E, González L, Campodónico L. Calcium supplementation to prevent hypertensive disorders of pregnancy. New England Journal of Medicine 1991; 325:1399-405

86. Belizán JM, Villar J, Zalazar A, Rojas L, Chan D, Bryce G F. Preliminary evidence of the effect of calcium supplementation on blood pressure in normal pregnant women. American Journal of Obstetrics and Gynecology 1983; 146:175-80.

87. Hofmeyr G Justus, Lawrie Theresa A, Atallah Álvaro N, Duley Lelia. Calcium supplementation during pregnancy for preventing hypertensive disorders and related problems. Cochrane Database of Systematic Reviews. In: *The Cochrane Library,* Issue 11, 2013

88. Belizan JM, Villar J, Repke J: The relationship between calcium intake and pregnancy-induced hypertension: up-to-date evidence. Am J Obstet Gynecol 1988, 158(4):898.

89. Belizán JM, Villar J, Self S, Pineda O, González I, Sainz E. The mediating role of the parathyroid gland in the effect of low calcium intake on blood pressure in the rat. Archivos Latinoamericanos de Nutrición 1984; 34:666-75
